# Effect of solution treatment on stress corrosion cracking behavior of an as-forged Mg-Zn-Y-Zr alloy

**DOI:** 10.1038/srep29471

**Published:** 2016-07-08

**Authors:** S. D. Wang, D. K. Xu, B. J. Wang, L. Y. Sheng, E. H. Han, C. Dong

**Affiliations:** 1 Key Laboratory of Nuclear Materials and Safety Assessment, Institute of Metal Research, Chinese Academy of Sciences, 62 Wencui Road, Shenyang 110016, China; 2 Laboratory of Materials Modification by Laser, Electron and Ion Beams, School of Materials Science and Engineering, Dalian University of Technology, Dalian 116024, China; 3 Peking University, Shenzhen Institute, Shenzhen Key Lab Human Tissue Regenerate & Repair, Shenzhen 518057, China

## Abstract

Effect of solid solution treatment (T4) on stress corrosion cracking (SCC) behavior of an as-forged Mg-6.7%Zn-1.3%Y-0.6%Zr (in wt.%) alloy has been investigated using slow strain rate tensile (SSRT) testing in 3.5 wt.% NaCl solution. The results demonstrated that the SCC susceptibility index (*I*_*SCC*_) of as-forged samples was 0.95 and its elongation-to-failure (*ε*_*f*_) was only 1.1%. After T4 treatment, the SCC resistance was remarkably improved. The *I*_*SCC*_ and *ε*_*f*_ values of T4 samples were 0.86 and 3.4%, respectively. Fractography and surface observation indicated that the stress corrosion cracking mode for as-forged samples was dominated by transgranular and partially intergranular morphology, whereas the cracking mode for T4 samples was transgranular. In both cases, the main cracking mechanism was associated with hydrogen embrittlement (HE). Through alleviating the corrosion attack of Mg matrix, the influence of HE on the SCC resistance of T4 samples can be greatly suppressed.

Due to the low density and high specific strength and stiffness, Mg alloys have recently received considerable attention for the applications in aerospace and automobile industries^1^,^2^,^3^,^4^. However, the low absolute strength and poor corrosion resistance greatly limited their industrial applications^5^,^6^,^7^,^8^,^9^,^10^. Recently, researchers reported that the I-phase (Mg_3_Zn_6_Y, icosahedral quasicrystal structure, quasi-periodically ordered) could enhance the mechanical properties of Mg-Zn-Y-(Zr) alloys at both ambient and elevated temperatures^11^,^12^,^13^. By combining powder metallurgy and hot extrusion processes, the fabricated I-phase containing Mg-Zn-Y alloys could have a yield stress of 410 MPa and an elongation of 12%^14^. Meanwhile, the yield strength of as-cast Mg-Zn-Y-Zr alloy could reach up to 450 MPa at room temperature depending on the volume fraction of I-phase^15^. Thus, the I-phase strengthened Mg-Zn-Y-(Zr) alloys have superior mechanical properties and could meet the mechanical requirements of structural components in automobile and aerospace industries. However, in real service conditions, the structural components often suffer from stress corrosion cracking (SCC) due to their exposure to the aggressive environment^16^,^17^. Generally, SCC is extremely dangerous, complicated and insidious in the real industries, which can cause unexpected and sudden fracture of structural components and lead to catastrophic accidents^16^. It has been reported that between 10 and 60 magnesium alloy components in aerospace applications alone suffered SCC failures each year^16^. Due to the increasing demand of Mg alloys in structural and automotive applications^16^,^18^,^19^, the deep understanding about their SCC behavior becomes more and more important.

It is well known that Mg dissolution in corrosive environment is an anodic reaction and accompanied by a cathodic reaction which is generally hydrogen evolution^20^,^21^. Thus, for Mg alloys, it is widely accepted that the SCC processes usually involve hydrogen embrittlement (HE)^16^,^17^,^18^,^21^,^22^, but the specific nature of the HE mechanism remains uncertain^20^. Several HE mechanisms have been proposed to explain the SCC behavior of Mg alloys, such as hydrogen enhanced de-cohesion (HEDE), hydrogen enhanced local plasticity (HELP), adsorption-induced dislocation emission (AIDE) and delayed hydride cracking (DHC)^23^,^24^,^25^,^26^,^27^,^28^,^29^,^30^,^31^,^32^. Detailed reviews of these mechanisms are provided elsewhere^16^,^33^,^34^,^35^,^36^,^37^. For the HEDE mechanism, it is proposed that hydrogen decreases the atomic bonding energy in the region ahead of the crack tip, where hydrogen accumulates by stress-assisted diffusion^23^,^24^. Then, it leads to tensile separation of adjacent metal atoms without requiring any plastic deformation. For the HELP mechanism, it is proposed that hydrogen enhances the mobility of dislocations, resulting in the formation of coalesced micro-voids^23^,^25^,^26^. The AIDE mechanism is associated with the dislocation emission, i.e. the metal-metal bonds could be weakened by the adsorption of hydrogen atoms at the crack tip and under the first few atomic layers^23^,^27^, which promotes crack growth by alternating slip on specific planes and then results in localised coalescence of micro-voids. The DHC mechanism involves repeated processes of stress-assisted diffusion of hydrogen to the region ahead of the crack tip, hydride (i.e. MgH_2_) formation and brittle fracture of hydride^28^,^29^,^30^,^31^,^32^. In the research about SCC behavior of an as-cast AZ91 Mg alloy, Chen *et al*. reported that hydrogen diffused to interior matrix, enriched and formed hydride at β phases and finally caused the cracking of β phases^38^. Additionally, previous work also reported that several mechanisms of HE can simultaneously occur depending on the material, strain rate and so on^23^,^28^,^39^.

Since Mg alloys would inevitably suffer from corrosion attack under corrosive environment^5^,^6^,^7^,^21^,^40^,^41^,^42^, corrosion severity should be considered for analyzing their SCC behavior. Moreover, the cathodic reaction of corrosion is the main source of hydrogen for causing HE^16^,^17^,^18^,^21^. To investigate the associated HE mechanisms in SCC, previous work mainly focused on the analysis of fracture morphologies^16^,^43^,^44^. However, the effect of corrosion severity on SCC behavior was usually neglected. Recently, it was reported that the corroded areas (dark areas) persistently existed on the surface of Mg alloys and acted as cathodes to sustain and enhance the hydrogen evolution rate^21^,^42^,^45^,^46^,^47^,^48^. This cathodic activation behavior could be catalysed by an enrichment of noble second phases or impurities in the dark corroded areas^41^,^45^,^46^,^49^,^50^,^51^, resulting in an accelerated hydrogen evolution rate. Moreover, the localised corrosion could provide a bare, film-free and active magnesium surface for ingress of hydrogen into the matrix. Then, the localised hydrogen build-up can embrittle the matrix (i.e. HE) and cause the subsequent cracking^16^,^17^,^20^,^22^,^36^,^52^. In previous work, Wang *et al*. reported that for an as-forged Mg-Zn-Y-Zr alloy in 0.1 M NaCl solution, MgZn_2_ precipitates existed in the alloy could induce severe localised corrosion^53^. After a solid solution treatment (T4), the corrosion resistance of the alloy was greatly enhanced^53^. Therefore, it can be predicted that due to the alleviated corrosion attack, the HE effect on SCC resistance of the T4 treated Mg-Zn-Y-Zr alloys should be reduced. However, so far, no relevant work about the effect of solution treatment on the SCC behavior of Mg-Zn-Y-Zr alloy can be referred. Moreover, whether the T4 treatment could improve the SCC resistance or not, it is still unknown.

In this work, the target is to investigate and compare the SCC behavior of an as-forged Mg-Zn-Y-Zr alloy before and after T4 treatment. Additionally, the underlying HE mechanism for both conditions will be deeply discussed.

## Results

### Microstructural characterization

**Figure 1:**
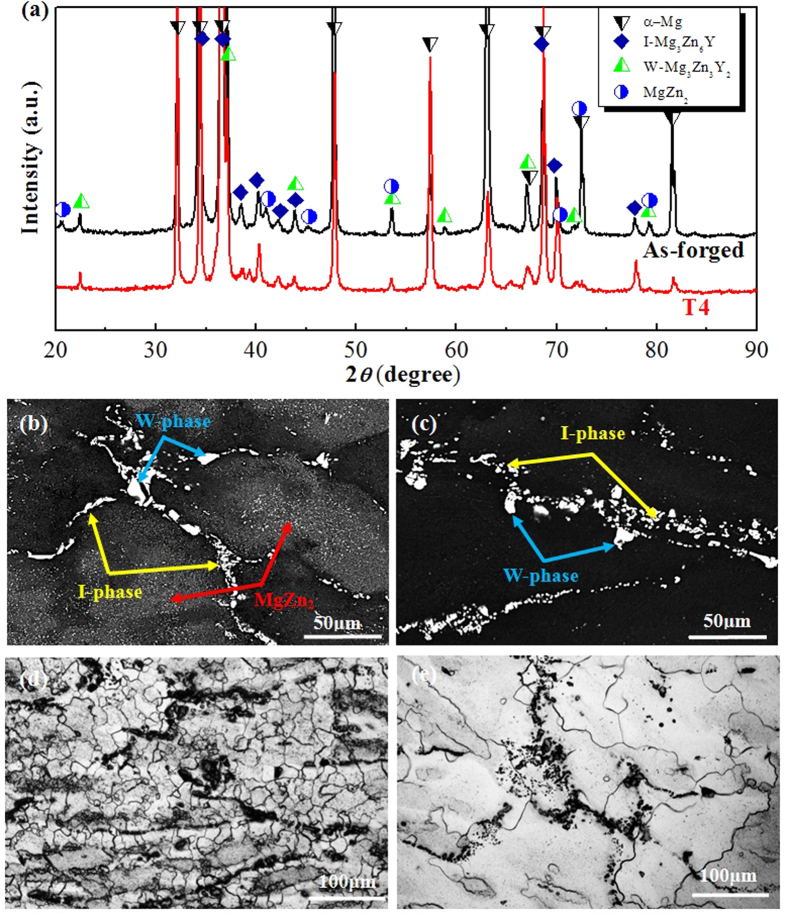
Microstructural analyses of the Mg-6.7%Zn-1.3%Y-0.6%Zr alloy: (**a**) XRD patterns of as-forged and T4 samples. Images (**b**,**c**) are the backscattered electron images of the alloy before and after T4 treatment. Images (**d**,**e**) are the etched microstructure of as-forged and T4 samples, respectively.

Microstructural analyses of the as-forged and T4-treated Mg-6.7%Zn-1.3%Y-0.6%Zr alloys are shown in Fig. 1. XRD patterns indicate that the as-forged alloy are mainly composed of I-phase, W-phase, MgZn_2_ and α-Mg (Fig. 1(a)).

After T4 treatment, strong diffraction peaks of I-phase, W-phase and α-Mg matrix were still detected. Therefore, the T4 treatment could not dissolve the I-phase and W-phase. However, the diffraction peaks corresponding to MgZn_2_ phases were basically disappearred, indicating their complete dissolution. To confirm it, the existed phases and their distribution before and after T4 treatment were observed and labeled (Fig. 1(b,c)). It reveals that after T4 treatment, no changes in the quantity, size, distribution and morphology of coarse I-phase and W-phase particles can be observed (Fig. 1(b,c)), which further proves their high melting temperatures. Moreover, a high density of MgZn_2_ precipitates could be observed in the as-forged sample (Fig. 1(b)), which was probably ascribed to the non-thermal holding during the hot forging process at elevated temperature. After T4 treatment, all the MgZn_2_ precipitates were dissolved (Fig. 1(c)). The grain structures of the alloy before and after T4 treatment are shown in Fig. 1(d,e). It can be seen that the average grain size of the as-forged sample was 20 μm. After T4 treatment, the grain structure was remarkably coarsened and the average grain size can reach 150 μm.

### Immersion and hydrogen evolution testing

**Figure 2:**
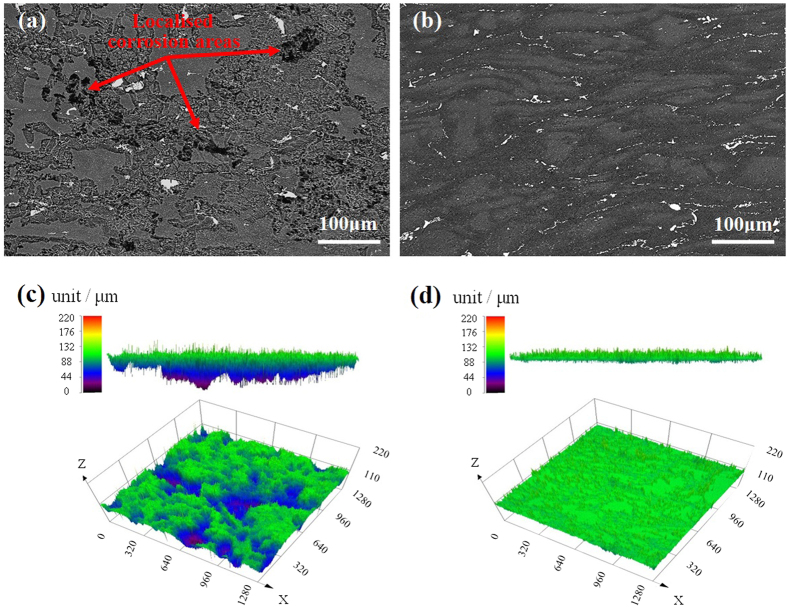
Surface observations after immersion in 3.5 wt.% NaCl solution for 6 h: (**a**,**b**) backscattered electron images of as-forged and T4 samples, respectively; (**c**,**d**) typical 3D CLSM images of as-forged and T4 samples, respectively.

To reflect the severity of corrosion attack, the corrosion morphologies of as-forged and T4 samples immersed in 3.5 wt.% NaCl solution were observed, as shown in Fig. 2. It can be seen that for the as-forged sample, several localised corrosion areas with a diameter of ~50 μm and severe corrosion grooves occurred after immersion for 6 h (Fig. 2(a)), whereas the corrosion attack on the T4 samples surface was quite weak and no severe localised corrosion areas can be observed (Fig. 2(b)). To compare the depth of corrosion attack, 3D CLSM images of corroded surfaces for samples immersed in NaCl solution for 6 h were observed (Fig. 2(c,d)). It revealed that localised corrosion areas in the as-forged samples were very large and their depth could reach up to 100 μm (Fig. 2(c)). However, for the T4 samples, severe localised corrosion areas could hardly be observed and only shallow corrosion grooves distribute on its surface (Fig. 2(d)).

**Figure 3:**
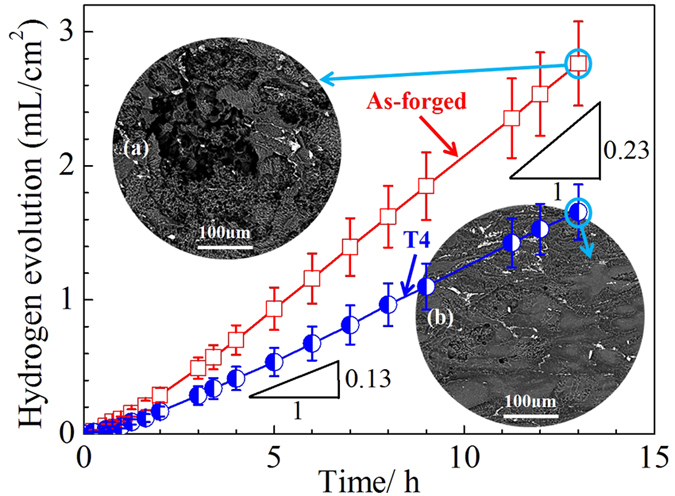
Hydrogen evolution of as-forged and T4 samples immersed in 3.5 wt.% NaCl solution for up to 13 h. The inserts are backscattered electron images of as-forged (**a**) and T4 (**b**) samples after hydrogen evolution test.

To further compare the corrosion resistance of two differently treated samples, the variation curves of hydrogen evolution versus immersion time are shown in Fig. 3. It revealed that the hydrogen evolution rate of as-forged samples was about 1.8 times as high as that of T4 samples. It is generally accepted that the hydrogen evolution rate can reflect the degradation rate of Mg alloys^54^. Therefore, it indicates that the corrosion attack of as-forged samples is much severer than that of T4 samples. To confirm it, the corroded surfaces of samples after hydrogen evolution testing were observed (Fig. 3(a,b)). It demonstrates that the localised corrosion occurred on the surface of as-forged samples is much severe than that of T4 samples, which is consistent with the trend in hydrogen evolution curves.

### E-T curves of the cathodic charging samples

**Figure 4:**
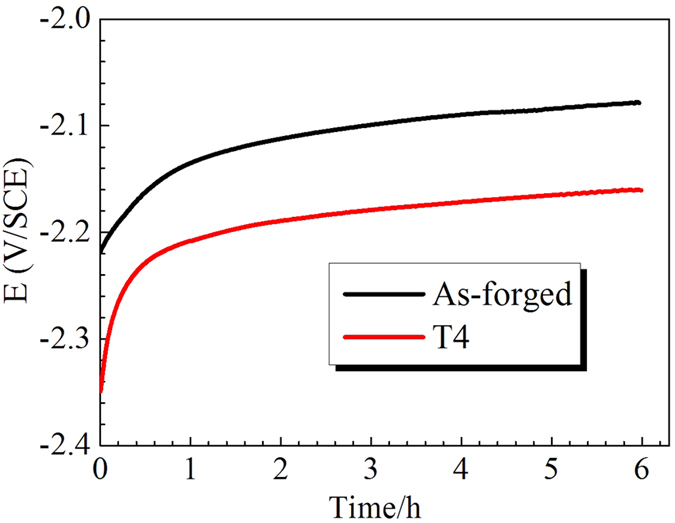
E-T curves of as-forged and T4 samples during the cathodic charging process in 3.5 wt.% NaCl solution for 6 h.

Figure 4 shows the potential changes versus cathodic charging hydrogen time curves of the pre-immersed samples. For differently treated samples, the potentials first rose rapidly and then tended to rise slowly. Previous work indicated that during cathodic charging process, the rise of potentials meant that oxide film (i.e. Mg(OH)_2_ or MgH_2_) formed on the sample surface and chemical stability of the samples was improved gradually^38^,^55^,^56^,^57^. Since T4 samples exhibited a higher growth rate of potential than as-forged samples, the chemical stability of T4 samples should be better than that of the as-forged samples^57^.

### Slow strain rate tensile (SSRT) testing

**Figure 5:**
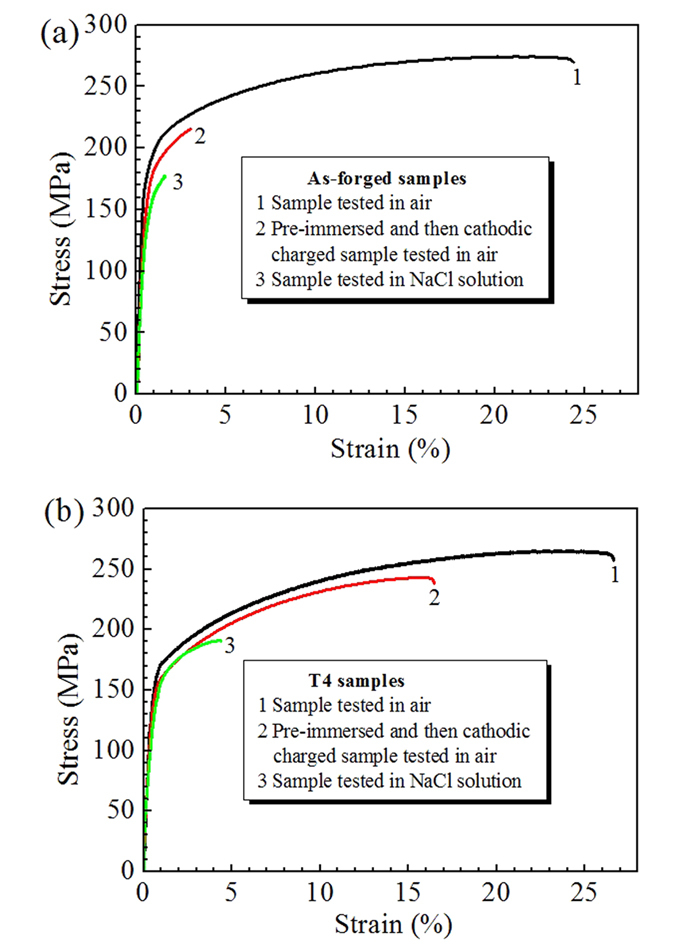
Stress-strain curves under different conditions: (**a**) as-forged and (**b**) T4 samples.

Figure 5 shows tensile curves of as-forged and T4 samples after different pre-treatments. To compare their mechanical properties, the change and decrease in terms of strength and plasticity are listed in Tables 1 and 2, respectively.

It can be seen that when tested in air, the difference of tensile properties between two differently treated samples is very slight. Among them, the yield strength (*YS*) and ultimate tensile strength (*UTS*) of the as-forged samples were 185 and 272 MPa, respectively. The *YS* and *UTS* of T4 samples were 160 and 265 MPa, respectively. Meanwhile, the plasticity of T4 samples was slightly higher than that of the as-forged samples (Table 1). When tested in 3.5 wt.% NaCl solution, the tensile properties significantly decreased when compared to those in air. It reveals that for the as-forged samples, the *YS* and *UTS* decrease to 141 and 177 MPa, respectively. Moreover, its elongation-to-failure (*ε*_*f*_) was only 1.1%. For the T4 samples, the *YS* and *UTS* decreased to 140 and 189 MPa, respectively. Additionally, the *ε*_*f*_ of T4 samples was 3.4%, which was approximately 3.1 times as high as that of the as-forged samples, indicating that T4 treatment can be helpful for improving the SCC resistance. After pre-immersion and cathodic charging, the as-forged samples showed a considerable loss of 11.3%, 20.6% and 88.3% in the *YS*, *UTS* and *ε*_*f*_ respectively when compared to those tested in air (Table 2). However, for the T4 samples, the relevant losses in the *YS*, *UTS* and *ε*_*f*_ were 6.9%, 8.3% and 38.6%, respectively (Table 2).

### Surface and cross-section observation

**Figure 6:**
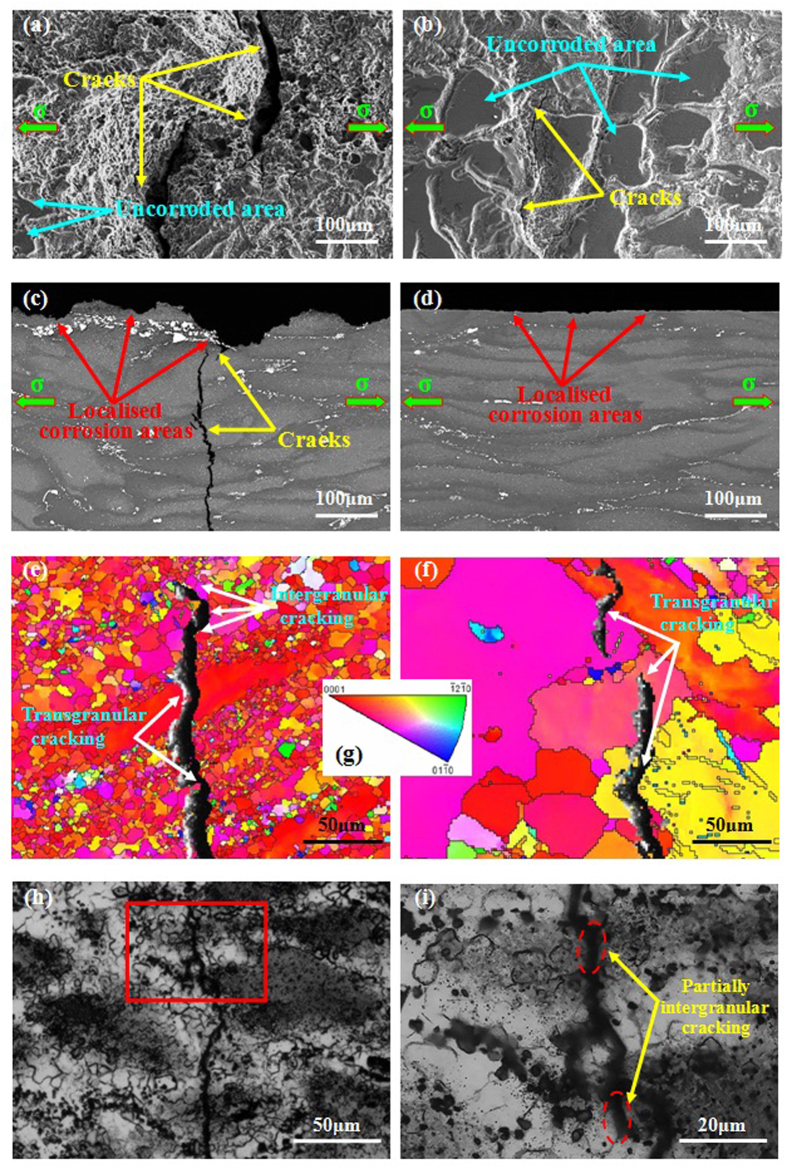
Microstructural analysis to the failed samples tensile tested in 3.5 wt.% NaCl solution: (**a**,**b**) secondary electron images of sample surfaces before and after T4 treatment; (**c**,**d**) backscattered electron imaging to cross-sectional surfaces of as-forged and T4 samples, respectively; (**e**,**f**) EBSD orientation maps to the grain structure of as-forged and T4 samples, respectively; Image (**g**) is the inverse pole figures reflecting the orientation relationship between the sample surfaces and crystallographic planes of grains; (**h**,**i**) optical images of the as-forged samples; Image (**i**) is high-magnification observation of the squared area in image (**h**).

To disclose the cracking mechanism, the gauge and cross-sectional surfaces of as-forged and T4 samples tensile tested in NaCl solution were observed, as shown in Fig. 6. It reveals that the as-forged samples have undergone severe localised corrosion on the surface (Fig. 6(a)). Meanwhile, large secondary cracks initiated at localised corrosion areas (Fig. 6(a)). On the contrary, the corrosion attack of T4 samples was relatively weak with shallow corrosion grooves on the surface (Fig. 6(b)). Moreover, secondary cracks were relatively small and mainly distributed in the region of corroded grooves (Fig. 6(b)). Based on their cross-sectional morphologies, it reveals that for the as-forged samples, the depth of localised corrosion areas can reach up to 100 μm and secondary cracks at or nearby the localised corrosion areas can be easily observed (Fig. 6(c)). For the T4 samples, the localised corrosion areas were relatively shallow (~5 μm) and secondary cracks can hardly be observed (Fig. 6(d)). To disclose their cracking mode, EBSD analyses to the cross-sectional surfaces of two samples were performed (Fig. 6(e–g)). Figure 6(e,f) present the inverse pole figure maps of two samples. The colors of grains in the maps correspond to the crystallographic axes of grains in the inverse pole figure, as shown in Fig. 6(g). It reveals that the cracking mode of the as-forged samples exhibits both transgranular and intergranular features, whereas for T4 samples, only transgranular cracking mode can be observed. It is to be noted that the samples were tilted at an angle of 70° for EBSD measurement, thus the cracks in EBSD orientation maps were different from their real morphologies. Moreover, the width of cracks is much larger than that of grain boundaries. Therefore, it would be very difficult to confirm interganular cracking on the surface of as-forged samples with fine grained structure. To reveal its cracking mode more directly, optical images of as-forged samples were provided, as shown in Fig. 6(h,i). It demonstrates that the cracking mode for as-forged samples was still predominated by transgranular. Due to the smaller grain size in as-forged samples, cracks can easily meet with grain boundaries. Thus, the partially intergranular cracking could possibly occur at the particular segment of some grain boundaries.

### Fractography

**Figure 7:**
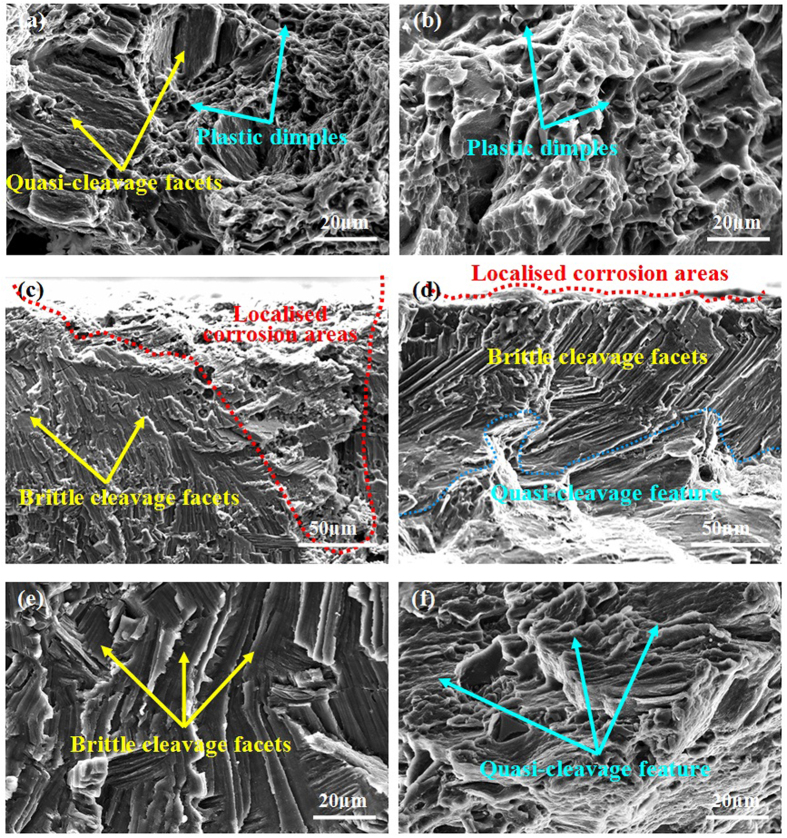
Fractography of samples under different conditions: (**a**,**b**) as-forged and T4 samples tested in air; (**c**,**d**) as-forged and T4 samples tested in 3.5 wt.% NaCl solution; (**e**,**f**) as-forged and T4 samples that pre-immersed plus cathodic charged tested in air.

The fracture surfaces of two samples tested in different conditions are shown in Fig. 7. It reveals that when tested in air, the fracture surface of as-forged samples has mixed-rupture characteristics of quasi-cleavage and plastic dimples, whereas the fracture surface of T4 samples is mainly composed of deep plastic dimples (Fig. 7(a,b)). When tested in NaCl solution, cracks preferentially occurred at localised corrosion areas for the as-forged samples (Fig. 7(c)). Moreover, brittle cleavage facets were observed, which was probably related to HE^20^. For T4 samples, the localised corrosion for cracking was relatively weak (Fig. 7(d)). Moreover, it showed brittle cleavage and quasi-cleavage features near and away from the margin of fracture surface, respectively. After being pre-immersed, cathodic charged and then tensile tested in air, brittle cleavage facets with fine steps can be observed for the as-forged samples, which was similar to those tested in NaCl solution (Fig. 7(e)). However, for the T4 samples, the fracture surface exhibited quasi-cleavage feature accompanied with many shallow plastic dimples (Fig. 7(f)).

## Discussion

To evaluate the SCC susceptibility of the alloy, the SCC susceptibility index (*I*_*SCC*_) was calculated by the loss in elongation^58^:


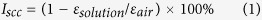


where *ε*_*solution*_ and *ε*_*air*_ are *ε*_*f*_ in 3.5 wt.% NaCl solution and air, respectively. When the value of *I*_*scc*_ approaches 1, it is assumed that the alloy is highly susceptible to SCC. Based on the Equation 1, the calculated *I*_*SCC*_ for the as-forged samples is 0.95, suggesting the as-forged samples is very susceptible to SCC in 3.5 wt.% NaCl solution. However, the calculated *I*_*SCC*_ for the T4 samples is 0.86, indicating that T4 treatment can enhance the SCC resistance of the investigated alloy. The mechanism for the improvement of SCC resistance due to T4 treatment will be discussed as follows.

Generally, the SCC processes occurred in Mg alloys usually involve HE because of the hydrogen from cathodic reaction of Mg dissolution^18^,^52^. However, there is a little consensus on a unified SCC mechanism^18^,^52^. To reveal the SCC mechanism of Mg alloys, it is better to understand the corrosion and hydrogen evolution mechanism^59^,^60^. Recently, Williams *et al*. demonstrated that persistent cathodes existed on the surface of dissolving Mg^42^. It indicates that the hydrogen gas comes from a persistent local cathodic reaction, which has been validated by Curioni *et al*. and Lebouil *et al*. using different methods^21^,^47^. Recently, this cathodic activation behavior was successively reported in commercial pure Mg and Mg alloys^45^,^46^,^48^. A phenomenological model proposed by Thomas *et al*. can well explain this behavior^41^. When Mg and Mg alloys were immersed in corrosive medium, a bilayered MgO/Mg(OH)_2_ film could form, appearing as a dark corroded area on the surface^41^. This dark corroded area gradually growed upon the surface and served as cathode to sustain and enhance the hydrogen evolution rate^41^. Williams *et al*. suggested that this dark corroded area could entrap noble impurities, which enhanced its catalytic activity towards the hydrogen evolution rate^46^. In the case of phase-containning Mg alloys, the cathodic activation was proposed to stem from noble second phases, which were known to be significantly much nobler than the α-Mg matrix^45^,^51^. Compared with the microstructural changes induced by T4 treatment, the main difference is that the as-forged samples contain a high density of MgZn_2_ precipitates, whereas all the MgZn_2_ precipitates are dissolved in the T4 samples (Fig. 1). Meanwhile, the grain size of T4 samples is 7.5 times larger than that of as-forged samples (Fig. 1). Birbilis *et al*. proposed that the finer grain structure can just have slight effect on the increase in corrosion resistance^61^. Thus, the poor corrosion resistance of as-forged samples is mainly ascribed to the MgZn_2_ precipitates. It has been reported that MgZn_2_ precipitates exhibit a cathodic behavior with respect to the Mg matrix due to their much nobler corrosion potential than that of α-Mg matrix^62^,^63^. Thus, corrosion attack could occur at the surrounding α-Mg matrix due to the existence of MgZn_2_ precipitates. Meanwhile, the MgZn_2_ precipitates may enrich in corroded areas, which would further promote cathodic activation and enhance hydrogen evolution^45^,^49^. Therefore, due to the dissolution of MgZn_2_ precipitates by T4 treatment, the hydrogen evolution rate of T4 samples is decreased (Fig. 3). Meanwhile, the corrosion attack of T4 samples can be suppressed for both statically immersed and tensile tested samples in NaCl solution (Figs 2 and 6).

**Figure 8:**
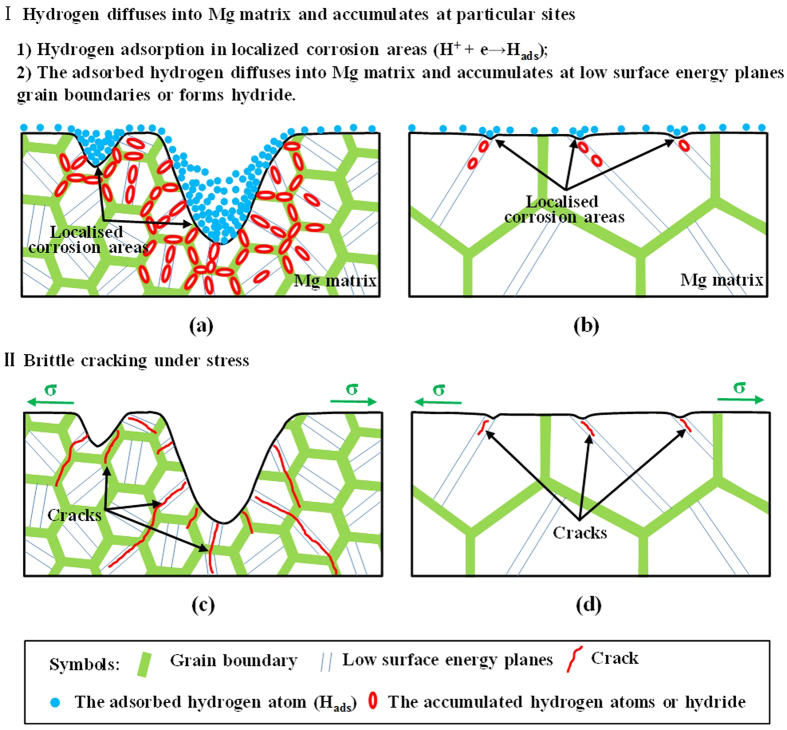
Schematic illustration of hydrogen embrittlement (HE) effect for the samples tensile tested in 3.5 wt.% NaCl solution: (**a**) accumulation of hydrogen atoms or formation of hydride and (**c**) HE-induced cracking of the as-forged samples; (**b**) accumulation of hydrogen atoms or formation of hydride and d) HE-induced cracking of the T4 samples.

Previous work indicated that hydrogen evolution was confined largely to localised corroded areas^45^,^49^. It was explained that the corroded areas were more cathodically active because second phases were exposed, or there was enrichment in noble alloying elements, or a less protective surface film was formed^45^,^49^. Since the severity of localised corrosion and hydrogen evolution rate are remarkably weakened by the T4 treatment, the concentration of hydrogen atoms at the bottom of localised corrosion areas of as-forged samples should be much higher than that of T4 samples. Figure 8 shows the schematic illustration of hydrogen embrittlement (HE) effect for two differently treated samples tensile tested in NaCl solution. For the as-forged samples, due to the cathodic activation effect, hydrogen atoms preferentially evolve and accumulate in the deep localised corrosion areas (Fig. 8(a)). Moreover, the concentration of hydrogen atoms especially at the bottom of localised corrosion areas could further increase with the immersion time, resulting in high concentration gradient of hydrogen atoms between localised corrosion areas and the surrounding Mg matrix^31^,^38^. Generally, low surface energy cystallographic planes (i.e. {0001}, {31-40}, {10-11}, {10-10}, {1-101}) and grain boundaries can provide preferential pathways for hydrogen diffusion in Mg alloys^27^,^43^,^64^. Thus, the evolved hydrogen atoms can easily diffuse into Mg matrix through those low surface energy planes and grain boundaries underneath the deep localised corrosion areas (Fig. 8(a)). Then, the diffused hydrogen atoms will gradually accumulate at these planes and grain boundaries. With prolonging the immersion time, the gradually accumulated hydrogen atoms could decrease the atomic bonding at these places^23^,^24^. When the stress is applied, these places will be preferentially act as the crack initiation sites (Fig. 8(c)). Thus, the cracking mode of as-forged samples is the mixture of transgranular and intergranular (Fig. 6(e)). On the contrary, for the T4 samples, the localised corrosion is remarkably alleviated and the hydrogen evolution rate is much lower than that of as-forged samples (Figs 3 and 6). Thus, the concentration of hydrogen atoms in the localised corrosion areas is relatively lower (Fig. 8(b)), resulting in a smaller concentration gradient between localised corrosion areas and the matrix. Moreover, due to the coarse grain structure of T4 samples, localised corrosion mainly occur within grains and can hardly meet grain boundaries (Fig. 8(b)). Then, the diffusion of hydrogen atoms preferentially occurs along the low surface energy planes and can be neglected at grain boundaries (Fig. 8(b)). Subsequently, high hydrogen buildup can only occur on the low surface energy planes. As a consequence, the dominant cracking mode of T4 samples is transgranular (Fig. 6(f)). Since the corrosion severity is slight and localised corrosion areas are quite small and shallow, the affected depth of localised hydrogen accumulation for stress corrosion cracking in the T4 samples should be much smaller than that in the as-forged samples (Fig. 8(d)), which is supported by the observations to fracture surfaces (Fig. 7(c,d)). The above HE process may be a reduction of atomic bonding at low surface energy planes and grain boundaries due to high hydrogen concentration at these places or the formation of a hydride. It has been reported that hydrogen atoms can react with Mg matrix to form stable hydride (i.e. MgH_2_) at room temperature^36^,^65^. In this case, some low surface energy planes (e.g. {2-203}) were reported to be the habit or cleavage planes of the hydride^16^,^30^,^35^. Since the segregation of hydrogen at grain boundaries has been widely reported, it is possible that hydride precipitation occurs at grain boundaries^36^,^38^,^66^. When the stress is applied, these places will preferentially act as the crack initiation sites due to the brittle cracking of hydride^29^,^44^. Based on the above analysis, the present model for HE mechanism is mainly related to the reduction in atomic bonding energy due to hydrogen accumulation or/and brittle cracking of the formed hydride. Since the corrosion severity of the alloy could be remarkably reduced by the T4 treatment, the improved SCC resistance of T4 samples should be mainly ascribed to a combined mechanisms of reduced anodic dissolution and weak HE.

## Methods

### Material preparation and treatments

The material used in the present work was an as-forged Mg-6.7%Zn-1.3%Y-0.6%Zr (in wt.%) alloy plate with a thickness of 50 mm and a deformation ratio of 5, which was prepared in the Magnesium Alloy Research Department of IMR, China. Sample pieces were cut from the plate for solution treatment at 300 °C for 1 h plus 400 °C for 2 h (T4) in an air furnace and then quenched into room temperature water.

### Microstructural analysis

After T4 treatment, samples were ground with SiC papers progressively down to 2000 grit, and finely polished to 1 μm finish with ethanol lubricant. Phase analysis was determined by X-ray diffraction (XRD) using a D/Max 2400 diffractometer with monochromatic Cu K_α_ radiation (wavelength: 0.154056 nm), a step size of 0.02° and a scan rate for data acquisition of 4°/min. Samples for optical observation were etched with an etchant of (1 ml nitric acid +1 ml oxalic acid+98 ml deionized water) and their average grain sizes were determined using the mean linear intercept method. Microstructures were observed by optical microscopy (OM) and scanning electron microscopy (SEM; XL30-FEG-ESEM).

### Immersion testing

To reflect the severity of corrosion attack, samples with dimension of 10 mm × 10 mm × 10 mm were immersed in 3.5 wt.% NaCl solution for 6 h at room temperature. The ratio of sample surface area (cm^2^) to the volume of NaCl solution (ml) was set to 1/50. Corrosion products after immersion were cleaned in a hot chromic acid bath consisting of 180 g/l CrO_3_^67^. Afterwards, surface morphologies of immersed samples were observed using confocal laser scanning microscopy (CLSM, OLYMPUS LEXT OLS4000).

### Hydrogen evolution testing

For hydrogen evolution experiment, samples with dimension of 10 mm × 10 mm × 10 mm were immersed in 3.5 wt.% NaCl solution which was open to the air for up to 13 h at a constant room temperature. The evolved hydrogen bubbles were collected into a burette above the corroded samples. No stirring or de-aerating was performed during immersion.

### Slow strain rate tensile (SSRT) testing

The SCC behavior of as-forged and T4 samples was investigated using the slow strain rate tensile (SSRT) method. Tensile samples with a gauge length of 25 mm, width of 6 mm and thickness of 3 mm were machined from the plate. The axial direction of tensile samples was parallel to the longitudinal direction (LD) of the plate. The surfaces of gauge sections were polished to a 1 μm finish and cleaned up using ethanol immediately before testing. For the SSRT test, samples were conducted on a MTS (858.01 M) testing machine at a constant strain rate of 1×10^−6^ s^−1^ at room temperature in 3.5 wt.% NaCl solution or air. In order to maintain the pH value near to sample surface, the gauge section of samples was immersed in an environmental cell and the solution was circulated from a tank with a volume of 5 L to the gauge section at a speed of about 167 ml/min using a circulating pump. After each test, the solution in the tank was changed. Moreover, tensile strain was recorded by an axial extensometer attached to the gauge length outside of the environmental cell. The detailed experimental setup for SSRT tests can be referred to the literature^68^. In order to investigate the effect of corrosion attack on HE, samples were pre-immersed in 3.5 wt.% NaCl solution for 6 h and then left in a desiccator for 28 days to allow desorption of any absorbed hydrogen^20^,^69^, and followed by cathodic charging in 3.5 wt.% NaCl solution for 6 h. To ensure the hydrogen evolution rates were the same for two differently treated samples, the galvanostatic charging mode at 27.8 mA/cm^2^ was applied. The potentials of the galvanostatic charged samples were recorded during the charging hydrogen process using an EG&G potentiostat model 273 and a classical three electrode cell with Pt counter electrode and saturated calomel reference electrode. Afterwards, these samples were immediately tensile tested in air. To ensure the reliability of measured data, at least three repeated measurements were carried out for each condition.

### Failure analysis

After testing, fracture surfaces of the tensile tested samples were cleaned in a hot chromic acid bath consisting of 180 g/l CrO_3_. Afterwards, surface and fracture characteristics were observed using SEM (XL30-FEG-ESEM). To characterize the cracking mode of tensile tested samples in 3.5 wt.% NaCl solution, their cross-sectional surfaces were examined by the EBSD method. EBSD measurements were carried out using a scanning electron microscopy (SEM; Hitachi S-3400N) equipped with an Oxford Instruments-HKL Channel 5 EBSD system at an accelerating voltage of 20 kV, a step size of 1.5 μm and a sample tilt angle of 70°.

## Additional Information

**How to cite this article**: Wang, S. D. *et al*. Effect of solution treatment on stress corrosion cracking behavior of an as-forged Mg-Zn-Y-Zr alloy. *Sci. Rep*. **6**, 29471; doi: 10.1038/srep29471 (2016).

## Figures and Tables

**Table 1:** Summary of the mechanical properties of as-forged and T4 samples tested in various conditions (The test in each condition was repeated three times, and standard deviation of the measured data was provided).

Conditions		*σ*_0.2_(MPa)	*UTS*(MPa)	*ε*_*f*__(%)_
As-forged	Tested in air	185.3 ± 7.0	272.3 ± 7.0	22.2 ± 1.9
	Pre-immersed, cathodic charged and tested in air	164.3 ± 5.0	216.3 ± 6.5	2.6 ± 0.4
	Tested in NaCl solution	141.3 ± 4.5	177.0 ± 5.0	1.1 ± 0.2
T4	Tested in air	159.7 ± 6.5	265.3 ± 7.5	24.1 ± 1.9
	Pre-immersed, cathodic charged and tested in air	148.7 ± 6.5	243.3 ± 7.5	14.8 ± 1.0
	Tested in NaCl solution	140.3 ± 7.5	189.3 ± 5.7	3.4 ± 0.4

**Table 2:** Decrease of mechanical properties for as-forged and T4 samples tested in various conditions as compared to those tested in air.

Conditions		Decrease of*σ*_0.2_(%)	Decrease of *UTS*(%)	Decrease of *ε*_*f*_(%)
As-forged	Pre-immersed, cathodic charged and tested in air	11.3	20.6	88.3
	Tested in NaCl solution	23.7	35.0	95.0
T4	Pre-immersed, cathodic charged and tested in air	6.9	8.3	38.6
	Tested in NaCl solution	12.1	28.6	85.9
